# Combining electroless ionisation mass spectrometry with solid-phase extraction for the direct analysis of beta-agonists in bovine urine

**DOI:** 10.1007/s00216-025-06019-3

**Published:** 2025-07-26

**Authors:** Sjors Rasker, Joris Schipperheijn, Stefan Kooij, Marco H. Blokland, Cees J. M. van Rijn, Ane Arrizabalaga-Larrañaga

**Affiliations:** 1 Wageningen Food Safety Research (WFSR), Part of Wageningen University & Research, P.O. Box 230, Wageningen, 6700 AE the Netherlands; 2 Van der Waals-Zeeman Institute, University of Amsterdam, Science Park 904, Amsterdam, the Netherlands

**Keywords:** Electroless ionisation, Ambient ionisation mass spectrometry, Solid-phase extraction, Bovine urine, Beta-agonists, ELI-MS

## Abstract

A novel method combining electroless ionisation mass spectrometry (ELI-MS) with solid-phase extraction (SPE) cartridges enables direct analysis of beta-agonist residues in bovine urine (SPE-ELI-MS). Using a syringe plunger and a battery-powered DIY syringe pump mounted on an XYZ-linear stage, rapid sample clean-up and precise positioning were achieved. Optimisation of spray solvent and nozzle placement improved performance, yielding high sensitivity, repeatability, linearity, and trueness with internal standard correction. This approach offers a simple, efficient solution for analysing organic residues in biological samples, with strong potential for future on-site applications.

**Graphical Abstract:**

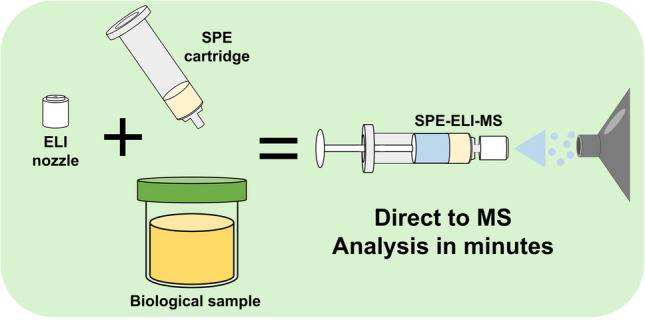

**Supplementary Information:**

The online version contains supplementary material available at 10.1007/s00216-025-06019-3.

## Introduction

Since the introduction of the first ambient ionisation mass spectrometry (AIMS) technique in 2004 [1], the field of ambient ionisation has grown rapidly [2–5]. The development of different AIMS techniques has greatly reduced the complexity and time required for sample analysis using mass spectrometry (MS) [6]. This has led to the application of AIMS techniques for screening purposes in food control, for example, for the analysis of antibiotics in animal products using paper spray ionisation (PSI) [7] and for the analysis of pesticides in vegetables using direct analysis in real time (DART) [8]. Additionally, AIMS techniques that offer additional selectivity by combining sample extraction with direct analysis have been developed, such as coated blade spray (CBS) [9]. This technique has been used to detect various food control–related substances, such as pesticides in oils [10] and veterinary drugs in muscle and kidney [11, 12]. Overall, the rapid developments in AIMS [6] over the years have led food safety researchers to explore and assess the many different options [e.g. 13–15] and developments in the field are still on-going [16, 17].

Recently, a novel ionisation interface has been developed, known as electroless ionisation (ELI) [18, 19]. ELI consists of a micro-fabricated spray nozzle that produces ions by the electro-kinetic interaction of the solvent with the nozzle surface. As such, it does not require a high voltage or gas supply, unlike other popular AIMS techniques in food analysis such as PSI, DART, and CBS. Additionally, its simplicity makes it easy, intuitive, and safe to use. The spray nozzle, which consists of a silicon chip with 1.9-µm-diameter holes, is mounted in an adapter with a Luer-slip type fitting, described in detail elsewhere [18, 19]. A charged spray can be produced by connecting the nozzle to a syringe filled with a spray solvent. ELI has been compared to the commonly used electrospray ionisation (ESI). It has already been used in case studies to analyse perfume through secondary ionisation and pesticide residues on citrus fruits [19]. However, it was shown that ELI is sensitive to the presence of salts, which can interfere with the charge build-up required for ionisation [18]. This means an additional sample clean-up step is required before the spraying and ionisation step to analyse biological matrices, which often contain relatively high concentrations of salts [20].

The beta-agonist class encompasses a wide range of structurally and functionally related substances that can be used for growth promotion in livestock rearing, which has been outlawed in the European Union by Council Directive 96/22/EC [21]. Monitoring the presence of beta-agonist residues is therefore relevant for food control. This is often performed in livestock urine, as expected levels in this matrix are relatively high [22], and it can be collected non-invasively. Generally, liquid chromatography tandem mass spectrometry (LC–MS/MS) methods are used for this purpose [e.g. 22, 23]; however, these methods are generally complex and time-consuming.

This work explores ELI’s capability as an AIMS technique for the analysis of bovine urine. First, a DIY battery-powered syringe pump was built specifically for SPE-ELI-MS. Next, the ELI nozzle’s spray solvent type and positioning relative to the MS-inlet were optimised. Then, to enable urine analysis with ELI, combining ELI with solid-phase extraction (SPE) cartridges was investigated to remove matrix components that interfere with ionisation. It is demonstrated that, by using SPE-ELI-MS, sample clean-up can be performed in minutes, and multiple beta-agonists, used as model substances in this work, can be readily detected in bovine urine. Additionally, the potential for quantitative analysis using internal standard correction is demonstrated.

## Experimental section

### Chemicals and reagents

LC–MS grade acetonitrile, methanol, and ethanol were purchased from Actu-All Chemicals (Oss, the Netherlands). Deionised water (produced with a Milli-Q Reference A + system; Merck KGaA, Darmstadt, Germany) was used for all experiments. Brombuterol, ractopamine, and zilpaterol were purchased from BVL Berlin (Berlin, Germany). Clenbuterol-HCl, salbutamol, salbutamol-d9, and brombuterol-d9 were purchased from Sigma-Aldrich (Amsterdam, the Netherlands). Clenbuterol-d9 was purchased from Witega (Berlin, Germany), ractopamine-d6-HCl was obtained from WFSR EU/CRL (Wageningen, the Netherlands), and zilpaterol-d7 was purchased from Toronto Research Chemicals (Toronto, Canada). Matrix experiments were performed with different batches of anonymised pooled blank bovine urine, which were provided by the Dutch Food Safety Authority (NVWA) and originated from the routine monitoring programme in the Netherlands. Standard solutions and matrices were stored at − 18 °C for the project’s duration.

### Instrumentation and software

The study was carried out using positive ionisation ELI nozzles (Ion Sprays, Amsterdam, the Netherlands) and a Xevo TQ-XS triple quadrupole mass spectrometer (Waters Corporation, MA, Milford, USA). The spray nozzle consists of a silicon nitride micro sieve chip with 85 precision-etched 1.9-μm-diameter pores contained in a polypropylene housing with a Luer-slip fitting, allowing it to be combined with Luer-slip type syringes and SPE cartridges. A DIY syringe pump built from 3D-printed and readily available, inexpensive structural and electronic parts (Inspired by another DIY syringe pump [25] controlled using a micro-controller (Elegoo Uno, Shenzhen, China) and powered with a conventional power bank was used for all ELI experiments. Interfacing with the MS was facilitated by attaching the syringe pump to a partially disassembled ionKey ionisation source (Waters Corporation, MA, Milford, USA); all 3D-printed parts were printed in PLA + (eSUN, Shenzhen, China) (see “Data availability” for the models).

During ELI experiments, the mass spectrometer settings were as follows: capillary voltage of 0.0 kV, source temperature of 150 °C, desolvation and cone gas flows were turned off, LM resolution 1 of 3, HM resolution 1 of 15, ion energy 1 of 0.5, LM resolution 2 of 3, HM resolution 2 of 15, ion energy 2 of 0.5. Values without units are instrument-specific values. The analytes and internal standards were monitored using the multiple reaction monitoring (MRM) transitions described in Supporting Information Table S1 (see Supporting Information Figure S1 for analyte chemical structures). Mass Lynx version 4.2 and Skyline version 24.1.0.199 were used for data analysis. StatGraphic 19 was used for the experimental design.

### Sample analysis

For the analysis of bovine urine, 1 cc Oasis HLB SPE cartridges (Waters Corporation, MA, Milford, USA) were combined with 3D-printed plungers. The seal between the plunger and the cartridge was ensured by using the rubber gasket from disposable 1-mL plastic syringes (Codan, Deventer, the Netherlands). The SPE cartridges were pre-conditioned using 1 mL of methanol and 1 mL of water. Next, 1 mL of sample was drawn up and eluted, followed by washing of the cartridges with 1 mL of water. After drying by nitrogen flow for 5 min, 0.5 mL of acetonitrile was drawn into the cartridge, after which the ELI nozzles were attached. Next, the SPE cartridge with the ELI nozzle attached is placed in a holder and then into the syringe pump. The syringe pump places the nozzle at a 90° angle to the MS-inlet, and exact positioning is facilitated by a XYZ-linear stage. For data collection, the MS was set to scan using the settings described earlier. The syringe pump is then turned on for approximately 30 s, after which the MS measurement is stopped. The ELI nozzle was replaced for every sample.

## Results and discussion

### Experimental set-up

**Fig. 1:**
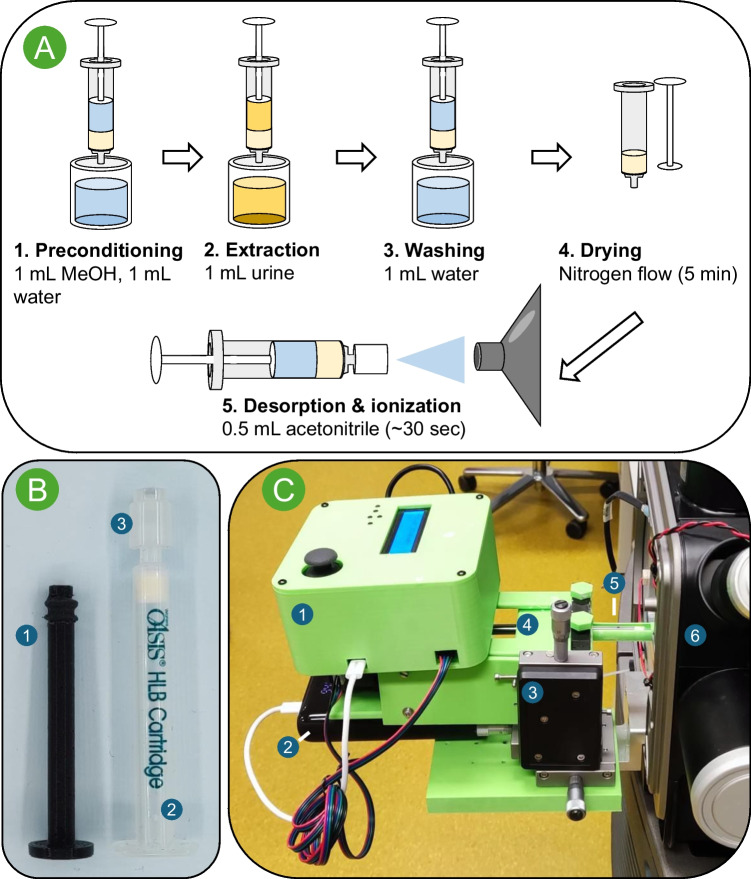
**A** Schematic overview of SPE-ELI-MS/MS workflow. **B** (1) 3D-printed syringe plunger and (2) SPE cartridge with (3) ELI nozzle attached. **C** Overview of the syringe pump attached to the mass spectrometer (MS) interface with (1) pump control unit, (2) power bank, (3) XYZ-stage, (4) syringe pump plunger push block, (5) SPE cartridge with ELI nozzle in holder at 90° angle to MS-inlet, and (6) stripped ion source to facilitate access to the MS interface

When an ELI nozzle is attached to a syringe, spraying and subsequent sample ionisation can be performed manually by pushing the syringe plunger (see Supporting Information Figure S2 for a full-scan comparison of ESI and ELI). However, manually maintaining an even flow rate with consistent positioning is difficult, so using a syringe pump is preferable. Therefore, a battery-powered syringe pump was built which was attached to the mass spectrometer interface using an XYZ-linear stage for positioning (Fig. 1C). The benefit of the syringe pump being battery-powered is that no external power source is required, which is also one of the benefits of ELI. Additionally, this enables the easy integration of the set-up with future portable MS systems for on-site analysis. The performance of the syringe pump was compared to that of a laboratory-grade syringe pump, which was determined to be similar (see Supporting Information Figure S3).

The ELI nozzle’s plastic housing enables coupling with conventional SPE cartridges for sample clean-up. SPE cartridges are normally used in combination with vacuum manifolds. However, they can also be used with plungers, allowing the user to draw a liquid sample into the SPE cartridge easily. A liquid sample can be passed through the SPE cartridge to extract the analyte of interest whilst interfering matrix components, such as dissolved salts, pass through. Using a washing step allows further sample clean-up. The analytes can then be desorbed by drawing in an appropriate solvent through the cartridge and ionised after the nozzle is attached (see Fig. 1A).

For this procedure, a plunger is required to manually fill and empty the cartridge subsequently for the syringe pump-powered spraying. To minimise the sample volume required for analysis, 1-cc SPE cartridges were used in this work. To the best of our knowledge, no plungers are available for this cartridge size, so plungers were designed and printed in-house, and a tight seal between the plunger and cartridge was ensured by using a rubber gasket taken from disposable syringes (see Fig. 1B).

### Optimisation

**Fig. 2:**
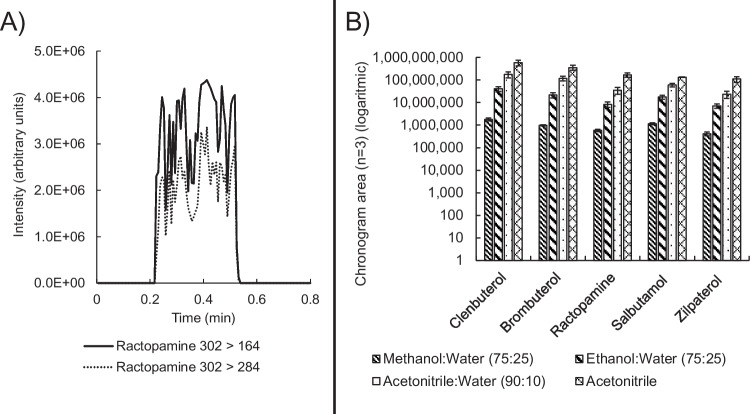
**A** Chronogram of ractopamine standard in acetonitrile. **B** Comparison of standards dissolved in different solvents (v/v) at 20 µg L^−1^. Error bars represent standard deviation (*n* = 3); *Y*-axis presented on a logarithmic scale

After establishing the experimental set-up for SPE-ELI-MS, the two main factors that could influence the results were optimised. The first factor was the solvent used for spraying and ionisation. Another consideration regarding the spray solvent was that it should be able to elute the analytes from the SPE cartridge. Therefore, three organic solvents commonly used in SPE and recommended for positive ionisation ELI [19] were tested, namely methanol, ethanol, and acetonitrile including 10 or 25% of water; additionally, acetonitrile without water was also tested. The positioning of the nozzle in relation to the MS-inlet was kept constant for each solvent at 1 cm to the side and 3 cm away from the inlet, with the flow set to 700 µL min^−1^. Figure 2A shows an example of an ELI-extracted chronogram obtained by spraying a ractopamine standard dissolved in acetonitrile. A chronogram visually presents the intensity of a mass spectrometric signal over the course of the measurement time. The measurement response was defined as the chronogram area of the most intense ion. Figure 2B shows the results of the spray solvent optimisation for all analytes. The highest response was found when using acetonitrile, followed by acetonitrile:water (90:10, v/v). The stark response differences observed between solvents are likely a result of the varying solvent-related charging efficiencies, although further experimentation would be required to investigate this more thoroughly.

**Fig. 3:**
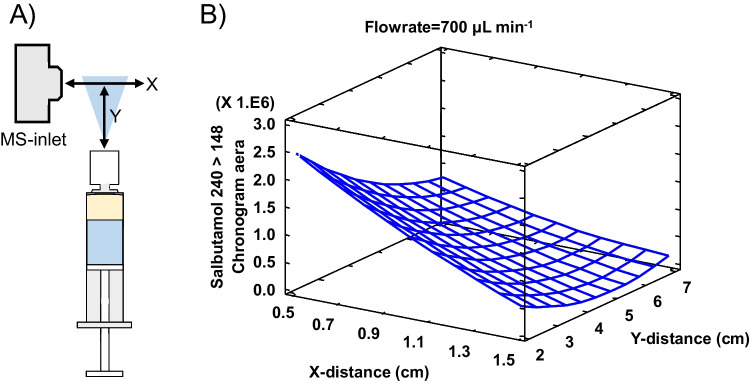
**A** Schematic top-down view of positioning measured from the middle of the MS-inlet. **B** Response surface of positioning for salbutamol at flow rate 700 µL min^−1^

After establishing the optimal spray solvent, the second factor, the positioning of the nozzle in relation to the MS-inlet, also considering the flow rate, was further optimised. Positioning was facilitated by the XYZ-linear stage. A systematic approach was used in the form of a three-factor Box-Behnken design (see Supporting Information Table S2 for details). The X distance and Y distance were defined as the distances between the nozzle and the MS-inlet (see Fig. 3A). The flow rate was also varied since this is known to affect the ionisation efficiency [19], and it was verified that a visually stable spray could be achieved with the flow rate set as low as 500 µL min^−1^ using the 1.9-µm-diameter, 85-hole ELI nozzles. The Z distance was not optimised and was kept constant at the level where the centre of the nozzle was in line with the middle of the MS-inlet. The angle of the nozzle to the inlet was maintained at 90° throughout all experiments to prevent overwhelming the desolvation capabilities of the inlet. Figure 3B contains an example response surface for salbutamol showing the signal intensity in relation to the X, Y positioning at a flow rate of 700 µL min^−1^. Each measurement was performed in duplicate, and the average response was used for the evaluation. Overall, the highest responses were found at an X, Y distance of 0.5 and 2 cm, respectively. However, varying positioning and flow rate effects were often statistically insignificant depending on the analyte (see Supporting Information Table S3). This insignificance is beneficial since it demonstrates that whilst the positioning does matter in some cases, there appears to be little benefit in strictly regulating the exact positioning and flow rate.

### Performance characteristics

**Fig. 4:**
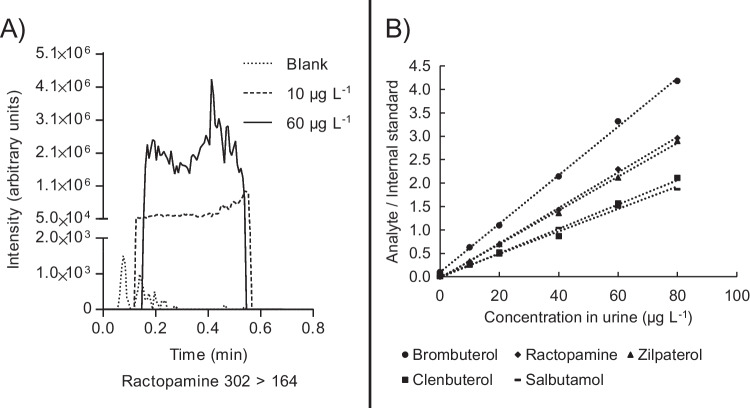
**A** Ractopamine chronograms in urine spiked at different levels. **B** Linearity of beta-agonists in bovine urine when using internal standard correction (*R*.^2^ between 0.991 and 0.999)

After optimising the experimental set-up, the performance of the procedure presented in Fig. 1A was tested for the analysis of several beta-agonists spiked in bovine urine as a proof-of-concept with regard to sensitivity, repeatability, linearity, and trueness. Figure 4A shows an example of chronograms obtained from analysing ractopamine in bovine urine. At 10 µg L^−1^, an apparent signal increase is observed compared to blank urine. When internal standard correction is applied, excellent repeatability is observed between measurements, with 3.9–4.8% values for three repeated measurements among the five included analytes. This is further illustrated by the assessment of the linearity of the corrected response in Fig. 4B, where a linear response (0.991–0.999) is found between 0 and 80 µg L^−1^ for all analytes. The trueness was assessed by analysing two other batches of bovine urine spiked at 60 µg L^−1^, and quantification was performed using the matrix fortified calibration curve (see Supporting Information Figure S4 for example chronograms). The calculated concentrations were between 57.7 and 70.3 µg L^−1^, corresponding to an average trueness of 106% (see Supporting Information Table S4 for a performance comparison to other analytical approaches). Interestingly, the analyte zilpaterol was only detectable in the bovine urine used for the calibration line and not in the other two bovine urines, indicating a matrix-mediated effect on the recovery, occurring either during extraction or ionisation.

## Conclusions

The feasibility of directly combining the novel AIMS technique, ELI, with SPE sample preparation was explored. Different spray solvents were tested, and the highest signal intensity was found for acetonitrile. The positioning of the ELI nozzle relative to the MS-inlet was optimised. Combining ELI with a SPE cartridge enabled quick and easy analysis, and it was demonstrated that using SPE-ELI-MS, beta-agonists could be extracted from bovine urine and subsequently analysed using MS. Different analyte concentrations added to urine resulted in different signal intensities linearly when applying internal standard correction, indicating that the method could potentially be used quantitatively. Combining ELI with SPE significantly broadens the range of applications for ELI, allowing easy sample clean-up of complex matrices combined with easy and quick analysis. This is especially promising in combination with future portable MS systems for on-site analysis with non-expert users.

## Supplementary Information

Below is the link to the electronic supplementary material.

Supplementary file1 (DOCX 1.02 MB)

## Data Availability

All models presented in this work and the technical details regarding the DIY syringe pump are available from 10.5281/zenodo.14823135. Other data is available upon request.
