# Supplementary material for: Combining electroless ionisation mass spectrometry with solid-phase extraction for the direct analysis of beta-agonists in bovine urine
Source: Anal Bioanal Chem. 2025 Jul 26;417(22):5019–25. doi: 10.1007/s00216-025-06019-3 (PMC12402018; doi:10.1007/s00216-025-06019-3)
Supplement: Supplementary file 1 — (DOCX 1.02 MB) [file 216_2025_6019_MOESM1_ESM.docx]

Supporting information

Combining electroless ionization mass spectrometry with solid phase extraction for the direct analysis of beta-agonists in bovine urine

Sjors Rasker^(1)*^, Joris Schipperheijn^(1)^, Stefan Kooij^(2)^, Marco H. Blokland^(1)^, Cees J.M. van Rijn ^(2)^, Ane Arrizabalaga-Larrañaga^(1)^

1. Wageningen Food Safety Research (WFSR), Part of Wageningen University &

Research, P.O. Box 230, 6700 AE Wageningen, the Netherlands

1. Van der Waals-Zeeman Institute, University of Amsterdam, Science Park 904, Amsterdam, the Netherlands

* Corresponding author: Sjors Rasker

E-mail: sjors.rasker@wur.nl

**Table of contents**

[Supporting Tables 3](#_Toc202882244)

[**Table S1:** Optimized MRM transitions for analytes and internal standards. 3](#_Toc202882245)

[**Table S2:** Experimental design for optimization of positioning. 3](#_Toc202882246)

[**Table S3:** Experimental design results for all analytes. 3](#_Toc202882247)

[**Table S4:** Comparison of performance characteristics 4](#_Toc202882248)

[Supporting Figures 6](#_Toc202882249)

[**Figure S1:** Chemical structures of included analytes 6](#_Toc202882250)

[**Figure S2:** Full-scan spectra comparison of ELI and ESI 7](#_Toc202882251)

[**Figure S3:** DIY Syringe pump in more detail and performance comparison. 8](#_Toc202882252)

[**Figure S4:** Ractopamine chronograms from two additional batches of bovine urine samples (A & B) spiked at 60 µg L^-1^. 9](#_Toc202882253)

# **Supporting Tables**

## **Table S1:** Optimized MRM transitions for analytes and internal standards.

| **Compound** | **Precursor ion (*m/z*)** | **Precursor adduct** | **Cone (V)** | **Product ion (*m/z*)** | **Possible fragments** | **Collision energy (eV)** |
| --- | --- | --- | --- | --- | --- | --- |
| Clenbuterol | 277 | [M+H]^+^ | 10 | 259 | [M+H–H₂O]^+^ | 10 |
|  |  |  | 10 | 203* | [M+H–H₂O–C₄H₈]^+^ | 15 |
| Brombuterol | 367 | [M+H]^+^ | 15 | 293* | [M+H–C₄H₁₀O]^+^ | 20 |
|  |  |  | 15 | 212 | [M+H–C₄H₁₀O–HBr]^+^ | 30 |
| Ractopamine | 302 | [M+H]^+^ | 30 | 164* | [HO–C₆H₄–C_2_H_4_–NH–CH(CH₃)]^+^ | 15 |
|  |  |  | 30 | 107 | [HO–C₆H₄–CH₂]^+^ | 30 |
| Salbutamol | 240 | [M+H]^+^ | 20 | 222 | [M+H–H₂O]^+^ | 10 |
|  |  |  | 20 | 148* | [M+H–2H₂O–C₄H₈]^+^ | 20 |
| Zilpaterol | 262 | [M+H]^+^ | 15 | 244* | [M+H–H₂O]^+^ | 10 |
|  |  |  | 15 | 185 | [M+H–C₆H₅N]^+^ | 25 |
| Clenbuterol-d_9_ (IS) | 286 | [M+H]^+^ | 5 | 204 | [M+H–H₂O–C₄D₈]^+^ | 15 |
| Brombuterol-d_9_ (IS) | 376 | [M+H]^+^ | 5 | 292 | [M+H–C₄D₁₀O]^+^ | 20 |
| Ractopamine-d_6_ (IS) | 308 | [M+H]^+^ | 10 | 168 | [D₆–HO–C₆H₄–C_2_D_4_–NH–CH(CH₃)]^+^ | 15 |
| Salbutamol-d_9_ (IS) | 249 | [M+H]^+^ | 20 | 231 | [M+H–H₂O]^+^ | 10 |
| Zilpaterol-d_7_ (IS) | 269 | [M+H]^+^ | 25 | 251 | [M+H–H₂O]^+^ | 15 |
| ** Used for quantification.* | | | | | | |

## **Table S2:** Experimental design for optimization of positioning.

| **Factor** | **Unit** | **Range and levels** | | |
| --- | --- | --- | --- | --- |
|  |  | **-1** | **0** | **+1** |
| X-distance | cm | 0.5 | 1 | 1.5 |
| Y-distance | cm | 2 | 4.5 | 7 |
| Flow | µL min^-1^ | 500 | 600 | 700 |

## **Table S3:** Experimental design results for all analytes.

| **Compound** | **Optimal x-distance (cm)** | **Optimal y-distance (cm)** | **Optimal flow rate (µL min^-1^)** |
| --- | --- | --- | --- |
| Clenbuterol | 0.500 | 2.0* | 516 |
| Brombuterol | 0.500 | 2.0* | 500 |
| Ractopamine | 0.500* | 2.0* | 500 |
| Salbutamol | 0.500* | 2.0* | 700 |
| Zilpaterol | 0.500* | 2.0* | 700 |

*Factors with an asterisk (*) indicate the significance of a factor for a compound.*

## **Table S4:** Comparison of performance characteristics

|  | Performance characteristic | SPE-ELI-MS | DART-MS [1] | LC-MS/MS [2] |
| --- | --- | --- | --- | --- |
| Brombuterol | Trueness (%) | 108 | N/A | 86 |
|  | Repeatability (%) | 3.9 | N/A | 8.67 |
|  | Correlation coefficient | 0.998 | N/A | 0.993 |
|  | Sensitivity (Limit of detection) (µg kg^-1^) | ≤ 10 | N/A | 0.03 |
| Ractopamine | Trueness (%) | 103 | N/A | 91.4 |
|  | Repeatability (%) | 3.9 | 28.3 | 11.3 |
|  | Correlation coefficient | 0.999 | 0.997 | 0.992 |
|  | Sensitivity (Limit of detection) (µg kg^-1^) | ≤ 10 | 3.1 ± 1.5 | 0.16 |
| Zilpaterol | Trueness (%) | 107 | N/A | 103.4 |
|  | Repeatability (%) | 4.7 | 28.9 | 13.81 |
|  | Correlation coefficient | 0.999 | 0.999 | 0.993 |
|  | Sensitivity (Limit of detection) (µg kg^-1^) | ≤ 10 | 19.5 ± 10.1 | 0.14 |
| Clenbuterol | Trueness (%) | N/A | N/A | 79 |
|  | Repeatability (%) | 1.5 | 33.4 | 5.25 |
|  | Correlation coefficient | 0.991 | 0.997 | 0.993 |
|  | Sensitivity (Limit of detection) (µg kg^-1^) | ≤ 10 | 1.2 ± 1.1 | 0.02 |
| Salbutamol | Trueness (%) | 96 | N/A | 97.4 |
|  | Repeatability (%) | 4.8 | 26.3 | 11.63 |
|  | Correlation coefficient | 0.995 | 0.993 | 0.992 |
|  | Sensitivity (Limit of detection) (µg kg^-1^) | ≤ 10 | 71.9 ± 39.4 | 0.17 |

[1] Shelver, W. L., Chakrabarty, S., & Smith, D. J. (2022). Rapid Screening Method for β-Adrenergic Agonist Residues Incurred in Animal Urine Using Direct Analysis in Real-Time Mass Spectrometry. *ACS Food Science & Technology*, *2*(1), 195-205. https://doi.org/10.1021/acsfoodscitech.1c00427

[2] Hajrulai-Musliu, Z., Uzunov, R., Jovanov, S., Musliu, D., Dimitrieska-Stojkovikj, E., Stojanovska-Dimzoska, B., Angeleska, A., Stojkovski, V., & Sasanya, J. J. (2023). Multi-class/residue method for determination of veterinary drug residues, mycotoxins and pesticide in urine using LC-MS/MS technique. *BMC Vet Res*, *19*(1), 156. https://doi.org/10.1186/s12917-023-03720-2

Compared to a recent screening method using direct analysis in real time (DART) [1], the SPE-ELI-MS approach demonstrates comparable sensitivity while showing comparatively better repeatability. Compared to a recent liquid chromatography-tandem mass spectrometry (LC-MS/MS) approach, the SPE-ELI-MS method's sensitivity is the main factor that needs to be improved to demonstrate comparable performance. It is expected that the sensitivity can be further improved by further optimization of the extraction and by further developing the experimental set-up.

Supporting Figures

## **Figure S1:** Chemical structures of included analytes

## **Figure S2:** Full-scan spectra comparison of ELI and ESI

Full-scan mass spectra recorded using a standard mixture of analytes at 100 µg L^-1^ in methanol, comparing A) ELI and B) ESI of the relevant *m/z* region with annotation of the major adducts of the included analytes.

## **Figure S3:** DIY Syringe pump in more detail and performance comparison.

The performance of the DIY syringe pump was compared to that of a laboratory-grade syringe pump (KD Scientific 789100) using a 12.2 mm diameter plastic syringe filled with water. The flow was set to the indicated levels, and the pump was turned on for 1 minute. The eluate was collected during this time and compared by weight to the theoretical amount of water that should be eluted at the set flow rate over this time. This experiment was performed in triplicate for each flow rate.

## **Figure S4:** Ractopamine chronograms from two additional batches of bovine urine samples (A & B) spiked at 60 µg L^-1^.
